# Orbital asymmetry degree: a potential ^99m^Tc-DTPA SPECT/CT imaging marker for diplopia in thyroid-associated ophthalmopathy

**DOI:** 10.3389/fendo.2026.1871573

**Published:** 2026-08-13

**Authors:** Xiaoan Pang, Jing Liang, Ziquan Zhou, Hengzhang Liang, Chen Yang, Zhixiao Wei

**Affiliations:** 1 Departmentof Nuclear Medicine, The First Affiliated Hospital of Guangxi Medical University, Nanning, China; 2 Department of Hospital Acquired Infection Management, The First Affiliated Hospital of Guangxi Medical University, Nanning, China

**Keywords:** ^99m^Tc-DTPA SPECT/CT, diplopia, imaging marker, inter-orbital inflammatory asymmetry, thyroid-associated ophthalmopathy

## Abstract

**Background:**

Diplopia is a common and clinically important manifestation of thyroid-associated ophthalmopathy (TAO), often related to asymmetric orbital involvement. This study investigated the association between orbital asymmetry degree (OAD), a novel quantitative imaging index derived from ^99m^Tc-DTPA SPECT/CT, and diplopia in patients with TAO.

**Methods:**

A1:1 matched case–control study was conducted, including 22 matched pairs of patients with TAO with or without diplopia. Patients were matched for sex, age, and smoking history. All participants underwent thyroid function testing, measurement of thyroid-related antibodies, and ^99m^Tc-DTPA SPECT/CT imaging. The maximum standardized uptake values (SUVmax) of the lacrimal glands, orbital soft tissues, and extraocular muscles were measured bilaterally. OAD was calculated from the absolute inter-orbital differences in SUVmax across these three orbital components and was used to quantify overall inter-orbital inflammatory asymmetry. Age-adjusted conditional logistic regression was performed to assess the associations of imaging and clinical variables, including OAD, the absolute inter-orbital difference in extraocular muscle SUVmax (dEMa), thyrotropin receptor antibody (TRAb), and CT-based mean extraocular muscle thickness, with diplopia. Full-sample ROC analysis and leave-one-pair-out cross-validation were used to evaluate model discriminatory performance.

**Results:**

After adjustment for age, both OAD (OR = 2.084, 95% CI: 1.040–4.174, *P* = 0.038) and dEMa(OR = 1.615, 95% CI: 1.044–2.497, *P* = 0.031) were significantly associated with diplopia, whereas TRAb and CT-based mean extraocular muscle thickness were not. In the full-sample ROC analysis, the OAD + age model showed the highest discriminatory performance (AUC = 0.940), compared with the dEMa + age model (AUC = 0.890) and the TRAb + age model (AUC = 0.785). Adding dEMa to the OAD + age model only slightly increased the AUC to 0.952, and Leave-one-pair-out cross-validation supported the relatively stronger internal performance of the OAD + age model, while the addition of dEMa did not provide meaningful improvement.

**Conclusions:**

Inter-orbital inflammatory asymmetry assessed by ^99m^Tc-DTPA SPECT/CT was significantly associated with diplopia in TAO.OAD showed relatively strong discriminatory performance and may represent a promising quantitative imaging marker. Further studies in larger prospective cohorts with independent external validation are needed to confirm these findings and clarify the clinical utility of OAD.

## 1 Introduction

Thyroid-associated ophthalmopathy (TAO), also known as Thyroid ophthalmopathy (TED) or Graves’ ophthalmopathy (GO), is a prevalent extrathyroidal manifestation of Graves’ disease, characterized by autoimmune inflammation of orbital soft tissues (1, 2). TAO exhibits a range of clinical features, such as proptosis, eyelid retraction, vision impairment, and diplopia with diplopia representing a clinically important functional complication (3, 4). In some cases, diplopia serves as the primary symptom in patients with TAO (5).

TAO-related diplopia is closely associated with extraocular muscle involvement. During the active phase of the disease, inflammation, edema, and subsequent fibrosis of the extraocular muscles may lead to restricted ocular motility and ocular misalignment, thereby resulting in diplopia (6, 7). However, the development of diplopia may not depend solely on the absolute degree of involvement of a single extraocular muscle. Rather, mechanical imbalance resulting from asymmetric inflammatory involvement between the two eyes may also play an important role in its pathogenesis (8, 9).At present, assessment of TAO activity mainly relies on the Clinical Activity Score (CAS) (10) and imaging modalities such as computed tomography (CT) and magnetic resonance imaging (MRI). Although CAS is simple and convenient to apply, it is inherently subjective and has limited sensitivity for detecting inflammation in the deep orbit, particularly within the extraocular muscles. CT (11) can demonstrate structural abnormalities such as extraocular muscle enlargement, whereas MRI, including STIR-based techniques, is an important tool for evaluating active orbital inflammation and increased orbital fat volume (12, 13). However, the potential contribution of quantitative inter-orbital inflammatory asymmetry to the functional manifestation of diplopia remains insufficiently explored.

^99m^Tc-diethylenetriaminepentaacetic acid (DTPA) is a sensitive functional imaging tracer of inflammatory activity. In inflamed tissues, increased capillary permeability allows ^99m^Tc-DTPA to extravasate from the vascular compartment into the interstitial space through compromised capillary walls, where it binds to extracellular peptides and aminoglycans (14). When combined with single-photon emission computed tomography/computed tomography (SPECT/CT), it enables precise anatomical localization and quantitative assessment of disease activity. Several studies have demonstrated the utility of ^99m^Tc-DTPA SPECT/CT in evaluating the inflammatory activity of TAO (15–19).Moreover, compared with traditional visual assessment or semi-quantitative methods, the maximum standardized uptake value (SUVmax) provides a more objective measure of local inflammatory activity (20).

Despite these advances, previous studies using ^99m^Tc-DTPA SPECT/CT in TAO have mainly emphasized disease activity and absolute or unilateral imaging findings. Therefore, rather than focusing on tracer uptake alone, the present study investigated whether SUVmax-based inter-orbital inflammatory asymmetry is associated with diplopia in patients with TAO, and evaluated orbital asymmetry degree (OAD), derived from multiple orbital compartments, as a novel quantitative imaging marker with potential relevance for diplopia risk stratification.

## 2 Material and methods

### 2.1 Data acquisition

This was a retrospective 1:1 matched case–control study conducted at the First Affiliated Hospital of Guangxi Medical University and included patients diagnosed with thyroid-associated ophthalmopathy (TAO) between September 2024 and March 2025. Patients with diplopia were assigned to the case group, whereas those without diplopia during the same period were assigned to the control group. Individual matching was performed at a 1:1 ratio according to sex, age (within 5 years), and smoking history (yes/no). A 1:1 matching strategy was adopted because, under the predefined matching criteria, not all cases had sufficient eligible controls to support a higher control-to-case ratio. When multiple eligible controls were available for a given case, one was randomly selected.

This study was conducted in accordance with the Declaration of Helsinki and was approved by the Ethics Committee of the First Affiliated Hospital of Guangxi Medical University (approval No. 2026-E0389).

#### 2.1.1 Inclusion and exclusion criteria

To ensure data reliability, the inclusion and exclusion criteria were predefined. Inclusion criteria were as follows (1): a definitive diagnosis of TAO based on the diagnostic criteria established by Bartley and Gorman in 1995 (21) (2); availability of high-quality ^99m^Tc-DTPA SPECT/CT images and complete clinical data; and (3) absence of vision-threatening complications or other orbital disorders. Clinical activity was independently assessed using the CAS (22)by two experienced nuclear medicine physicians. In cases of disagreement, a third experienced nuclear medicine physician reviewed the case, and the final score was determined by consensus. The severity of TAO was determined by ophthalmologists during routine clinical consultation.

Patients were excluded if they had other ocular or orbital diseases that could affect the orbit or visual function, including retrobulbar space-occupying lesions, diabetic retinopathy, hypertensive fundus disease, or other autoimmune disorders. Patients with a history of orbital decompression surgery or orbital radiotherapy, as well as those younger than 18 years, were also excluded.

#### 2.1.2 Judgment of binocular diplopia

Diplopia was determined through routine clinical ophthalmologic assessment. The attending physician first asked each patient about TAO-related symptoms and performed an initial ocular motility examination in nine gaze positions under fixed head position. Patients were then referred for ophthalmologic consultation, where diplopia was confirmed and documented in the medical record. Only patients with binocular diplopia were included in the diplopia group. This case definition was intended to capture clinically evident binocular diplopia associated with ocular misalignment or motility disturbance.

A total of 160 patient records were initially screened for eligibility. After exclusion of 35 patients with incomplete clinical data or poor-quality SPECT/CT images, 3 with a history of orbital decompression or other ocular surgery, and 1 with prior orbital radiotherapy, 121 eligible patients remained. Among these, 22 had diplopia and 99 did not. A 1:1 individual matching procedure was then performed, and 22 patients without diplopia were selected from the 99 eligible controls to match the 22 diplopia cases, resulting in 22 matched case–control pairs for the final analysis.

### 2.2 SPECT/CT acquisition protocol

Orbital SPECT/CT imaging was conducted 20 minutes post-injection of 740 MBq (20 mCi) of ^99m^Tc-DTPA into the elbow vein. The patient assumed a supine position on the examination table with the head immobilized and eyes shut. Initially, SPECT tomography was acquired using a low-energy high-resolution collimator. The acquisition matrix was 128×128 with a magnification factor of 1. Each frame had a 20-second acquisition time, totaling 60 frames. Subsequently, a CT scan was performed with a slice thickness of 0.625mm, a pitch ratio of 1:1, a tube voltage of 140 kV, and a tube current of 260 mA. The activity of ^99m^Tc-DTPA in the syringe before and after injection, and the time of measurement were all documented. Following data acquisition, the Xeleris3.1 system (GE Company, USA) was utilized for post-processing to produce a range of cross-sectional orbital CT images, SPECT images, and SPECT/CT fusion images. Furthermore, SPECT images underwent reconstruction employing an iterative ordered subset expectation maximization (OSEM) algorithm with 2 iterations and 10 subsets, incorporating CT-based attenuation correction, scatter correction, and resolution recovery within the Xeleris3.1 system. A post-reconstruction Butterworth filter with a critical frequency of 0.48 and power of 10 was applied.

### 2.3 Indices of the quantitative SPECT/CT

#### 2.3.1 Calculation of SUVmax

Information on acquisition, such as camera sensitivity, patient demographics, activities in both full and empty syringes, administration and scan times, and tracer details, was entered into the system. Subsequently, the volumes of interest (VOIs) for the lacrimal glands(LG), orbital soft tissues(OST), and extraocular muscles(EM) were automatically delineated on SPECT images using an NM threshold of 0.4. The system could then automatically compute SUVmax values.

Note: for each eye, extraocular muscle SUVmax was defined as the maximum SUVmax among the superior rectus, inferior rectus, medial rectus, and lateral rectus muscles.

#### 2.3.2 Quantitative imaging indices related to diplopia

To quantify inter-orbital inflammatory asymmetry, dLGa, dOSTa, and dEMa were defined as the absolute differences in bilateral SUVmax values for the lacrimal glands, orbital soft tissues, and extraocular muscles, respectively, with each value multiplied by 10 to facilitate numerical presentation and interpretation. Specifically,


dLGa=∣SUVmax_LG,right-SUVmax_LG,left∣×10

dOSTa=∣SUVmax_OST,right−SUVmax_OST,left∣×10, and

dEMa=∣SUVmax_EM,right−SUVmax_EM,left∣×10

SUVmax_LG,right/left, SUVmax_OST,right/left, and SUVmax_EM,right/left denote the SUVmax values of the right and left lacrimal glands, orbital soft tissues, and extraocular muscles, respectively.

OAD was then calculated as the Euclidean distance from the three-dimensional coordinate Z=(dLGa,dOSTa,dEMa) to the origin (0,0,0), providing a quantitative imaging measure of inter-orbital inflammatory asymmetry.


$$OAD=OZ=\sqrt{dLGa^{2}+dOSTa^{2}+dEMa^{2}}$$

Mean extraocular muscle thickness was also measured for each orbit on the CT component of SPECT/CT as the average of the maximal short-axis diameters of the superior, inferior, medial, and lateral rectus muscle bellies on coronal images. The absolute inter-orbital difference in mean extraocular muscle thickness was defined as the absolute difference between the left and right orbital mean values.

### 2.4 Statistical analysis

This study employed a 1:1 paired case-control design, and all statistical analyses were conducted using R software (version 4.5.2). Continuous variables are presented as mean ± standard deviation or median (interquartile range), as appropriate, and categorical variables as frequency and percentage. Between-group comparisons were performed using paired t-tests or Wilcoxon signed-rank tests.

Variables showing statistical significance were entered into age-adjusted conditional logistic regression models, with matched pair identifier used as the stratification variable. Results are reported as odds ratios (ORs) and 95% confidence intervals (CIs). ROC curves and AUC values were generated from the fitted models to assess discriminatory performance for diplopia based on the full study sample. Optimal cutoff values were determined using the maximum Youden index, and the corresponding sensitivity and specificity were reported. To further assess model robustness, internal validation was performed using leave-one-pair-out cross-validation while preserving the matched case–control structure. Model performance was assessed according to whether the diplopia case received a higher model-derived linear predictor value than the matched control within each held-out pair. Statistical significance was set at *P* < 0.05.

## 3 Result

### 3.1 Comparison of demographic and thyroid-related clinical characteristics

The results indicate that despite utilizing range matching for age in this study, which was not an exact match, a significant age difference persisted between the two groups. Additionally, there was a discernible distinction in TRAB levels between the case group (diplopia) and the control group (non-diplopia) (all *P* < 0.05). However, no statistically significant differences were observed in the levels of FT3, FT4, TSH, TPOAb, TGAb, dCASa and severity degree in both eyes (all *P*> 0.05) (**Table 1**).

**Table 1:** Comparison of demographic and thyroid-related clinical characteristics between the case and control groups.

Variable	Case group(n=22)	Control group(n=22)	Statistic	*P* Value
Age(year)	36.55 ± 17.15	37.55 ± 16.66	-2.291	0.032
FT3(pmol/l)	35.68 ± 12.44	35.63 ± 13.14	0.017	0.987
FT4(pmol/l)	61.69 ± 17.09	60.87 ± 19.01	0.170	0.867
TSH(mIU/I)	15.1 ± 14.63	12.22 ± 12.75	0.703	0.489
TPOAb(IU/mI)	289.5 (164.75, 646.5)*	316.5 (101.62, 924.25)*	140	0.673
TRAb(IU/I)	24.54 ± 12.82	16 ± 12.74	2.272	0.034
TGAb(%)	32.02 ± 24.93	35.8 ± 27.11	-0.655	0.520
dCASa	0 (0,0)	0 (0,0)	1	1
Severity degree of right eye^#^	1.5 (1.0–2.0)	1.0 (1.0–1.75)	90.0	0.079
Severity degree of left eye^#^	1.0 (1.0–2.0)	1.0 (1.0–1.75)	77.5	0.108

*Data are presented as median (interquartile range) or else presented as mean ± standard deviation.

dCASa=the absolute inter-orbital difference in Clinical Activity Score

#Severity degree was coded as 0 = no ophthalmopathy,1 = mild disease,and 2 = moderate-to-severe disease. Data are presented as median (interquartile range). Paired comparisons were performed using the Wilcoxon signed-rank test.

### 3.2 Comparison of quantitative imaging indices

The comparative analysis of the two groups revealed that the OAD level in the case group was significantly higher than that in the control group (*P* < 0.05). In contrast, while dLGa, dOSTa, and dEMa were elevated in the case group compared to the control group, these differences did not reach statistical significance (all *P* > 0.05). Notably, the *P* values for dOSTa and dEMa were 0.066 and 0.077, respectively, indicating a potential trend toward significance (**Table 2**).

**Table 2:** Comparison of imaging indices between case and control group.

Variable	Case group(n=22)	Control group(n=22)	Statistic	*P* Value
dLGa	1.7 (0.95, 3.25)	1.15 (0.91, 2.3)	174	0.127
dOSTa	2.37 ± 2.05*	1.54 ± 1.03*	1.939	0.066
dEMa	2.86 ± 2.58*	1.61 ± 1.23*	1.863	0.077
OAD	4.92 (2.58, 8.57)	2.79 (2.02, 3.55)	217	0.004

*Data are presented as mean ± standard deviation,or else presented as median (interquartile range).

dLGa=absolute inter-orbital difference in lacrimal gland SUVmax× 10.

dOSTa=absolute inter-orbital difference in orbital soft tissue SUVmax× 10.

dEMa=absolute inter-orbital difference in extraocular muscle SUVmax× 10.

OAD=orbital asymmetry degree.

SUVmax values of the left and right extraocular muscles were also compared between groups and showed no significant differences [SUVmax_EM,left, *P* = 0.894; SUVmax_EM,right, *P* = 0.987]. For CT-based measurements, mean extraocular muscle thickness of the left orbit was slightly greater in the case group than in the control group [3.318 (2.873, 3.833) vs. 3.054 (2.768, 3.420), *P* = 0.048], whereas the difference in mean thickness of the right orbit did not reach statistical significance (*P* = 0.079). The absolute inter-orbital difference in mean extraocular muscle thickness was also not significantly different between groups (*P* = 0.243) (**Supplementary Table 1**).

### 3.3 Age-adjusted conditional logistic regression analysis of factors associated with TAO related diplopia

Based on the between-group comparisons of demographic, thyroid function, antibody, and imaging indices, variables showing statistically significant differences were selected for further analysis. Each of these variables was then individually entered into an age-adjusted conditional logistic regression model to assess its association with diplopia. In addition, given the central role of extraocular muscle involvement in the pathogenesis of TAO-related diplopia, dEMa and CT-based mean extraocular muscle thickness were also included in the regression analysis, although some of these variables showed only borderline significance in the pairwise comparison.

The regression results demonstrated that, after adjustment for age, OAD (OR = 2.084, 95% CI: 1.040–4.174, *P* = 0.038) was significantly associated with diplopia. Likewise, dEMa (OR = 1.615, 95% CI: 1.044–2.497, *P* = 0.031) was also significantly associated with diplopia. In contrast, TRAb (OR = 0.795, 95% CI: 0.381–1.657, *P* = 0.540) and CT-based mean extraocular muscle thickness of the left and right orbits were not significantly associated with diplopia after age adjustment (**Table 3**; **Supplementary Table 2**).

**Table 3:** Age-adjusted conditional logistic regression analysis of OAD, dEMa and TRAB in patients with TAO.

Variable	Age-adjusted OR	95%CI	*P* Value
OAD	2.084	1.040~4.174	0.038
dEMa	1.615	1.044~2.497	0.031
TRAb	0.795	0.381~1.657	0.540

dEMa=absolute inter-orbital difference in extraocular muscle SUVmax× 10.

OAD= orbital asymmetry degree.

### 3.4 ROC analysis and comparison of discriminatory performance across predictive models

**Figure 1:**
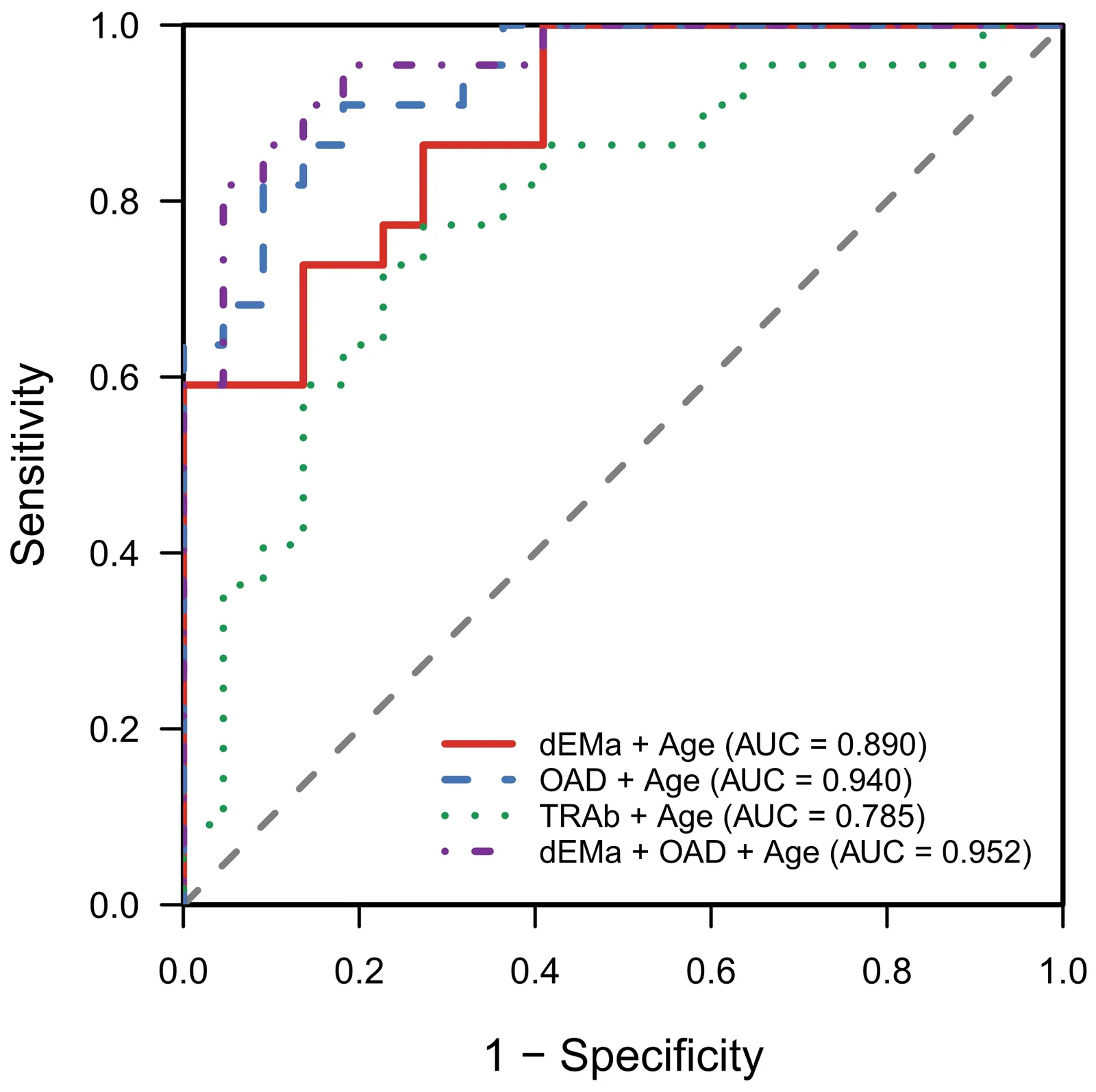
Receiver operating characteristic (ROC) curves of the four age-adjusted predictive models.

In the full-sample analysis, the ROC curves and AUC values shown in **Table 4** and **Figure 1** were derived from the same dataset used for model fitting. Among the three age-adjusted univariate models, the OAD + age model showed the highest discriminatory performance (AUC = 0.940, 95% CI: 0.878–1.000), compared with the dEMa + age model (AUC = 0.890, 95% CI: 0.800–0.981) and the TRAb + age model (AUC = 0.785, 95% CI: 0.646–0.925) (all *P* < 0.001). Although the combined OAD + dEMa + age model slightly increased the AUC to 0.952 (95% CI: 0.897–1.000), both predictors became statistically non-significant when entered together (**Table 4**; **Supplementary Table 3**).

**Table 4:** ROC analysis based on the full study sample and comparison of discriminatory performance among predictive models.

Model	AUC	95%CI	Cutoff*	Youden_Index	*P* value	Sensitivity	Specificity	PPV	NPV
OAD + Age	0.940	0.940 (0.878-1.000)	-0.191	0.727	<0.001	0.909	0.818	0.833	0.900
dEMa+ Age	0.890	0.890 (0.800-0.981)	-0.648	0.591	<0.001	1.000	0.591	0.710	1.000
TRAb + Age	0.785	0.785 (0.646-0.925)	-0.046	0.500	<0.001	0.773	0.727	0.739	0.762
OAD+ dEMa+ Age	0.952	0.952 (0.897-1.000)	-0.407	0.773	<0.001	0.955	0.818	0.840	0.947

PPV=positive predictive value.

NPV=negative predictive value

*Cutoff values represent thresholds of the model-derived linear predictor from ROC analysis rather than raw cutoff values

Leave-one-pair-out cross-validation was additionally performed to assess model robustness in this small matched cohort. Using this pair-level internal validation approach, the OAD + age model correctly ranked the diplopia case above the matched control in 18 of 22 held-out pairs (81.8%), compared with 16 of 22 pairs (72.7%) for the dEMa + age model and 15 of 22 pairs (68.2%) for the TRAb + age model. The combined OAD + dEMa + age model also correctly ranked 18 of 22 pairs (81.8%), indicating no meaningful improvement over the OAD + age model alone (**Table 5**).

**Table 5:** Leave-one-pair-out cross-validation results of the age-adjusted predictive models.

Model	Correctly ranked* pairs/total pairs	Pair-level accuracy (%)
OAD + Age	18/22	81.8
dEMa + age	16/22	72.7
TRAb + age	15/22	68.2
OAD + dEMa + age	18/22	81.8

*A correct ranking was defined as a higher model-derived linear predictor value for the diplopia case than for the matched control within each held-out pair.

## 4 Discussion

In this study, we investigated the association between diplopia in patients with TAO and OAD, a quantitative imaging index derived from the SUVmax values of the bilateral lacrimal glands, orbital soft tissues, and extraocular muscles on ^99m^Tc-DTPA SPECT/CT. Because diplopia was present in only a minority of the eligible TAO patients (22 of 121), a 1:1 matched case–control design was adopted to address the substantial imbalance between patients with and without diplopia. By matching on sex, age, and smoking history, this approach improved comparability between groups and reduced potential baseline confounding. Although matching was performed at the design stage, a residual age difference remained, indicating that age could still act as a residual confounder and should therefore be adjusted for the subsequent regression analyses.

Age-adjusted conditional logistic regression analyses showed that both OAD and dEMa were significantly associated with diplopia (OR = 2.084 and 1.615, respectively). In contrast, SUVmax values of the left and right extraocular muscles were similar between groups, and CT-based mean extraocular muscle thickness showed no consistent inter-orbital asymmetry or significant association with diplopia after age adjustment. TRAb was also not significantly associated with diplopia, despite previous reports (12) identifying serum TRAb as an independent risk factor for TAO-related diplopia. One possible explanation is that diplopia is primarily a localized orbital manifestation, and local orbital inflammatory changes may therefore be more directly related to its occurrence than side-specific uptake, structural enlargement, or systemic autoantibody levels. In addition, the relatively small sample size and differences in study design and patient characteristics may also have contributed to this inconsistency. Overall, these findings support our hypothesis that local inter-orbital inflammatory asymmetry may be more directly related to diplopia, consistent with the recognized asymmetric presentation of Graves’ orbitopathy (23). Notably, dCASa was close to zero in both groups, suggesting that CAS-based clinical assessment may be less sensitive to inter-orbital imbalance relevant to diplopia than SUVmax-based inflammatory asymmetry indices such as OAD and dEMa.

Importantly, the present study focused not simply on orbital tracer uptake, but on inter-orbital inflammatory asymmetry quantified by OAD, which may be more relevant to diplopia than uptake intensity alone. OAD represents a novel and objective quantitative imaging marker for characterizing inter-orbital inflammatory asymmetry in TAO. By integrating the absolute inter-orbital differences in SUVmax of the lacrimal glands, orbital soft tissues, and extraocular muscles into a three-dimensional coordinate [Z = (dLGa, dOSTa, dEMa)], OAD captures the orbital imbalance in inflammatory activity between the two eyes. In patients without diplopia, this coordinate would be expected to lie closer to the origin (0, 0, 0), whereas a greater Euclidean distance from the origin indicates a higher degree of inflammatory asymmetry. Importantly, OAD not only combines multiple asymmetry measures into a single quantitative marker, but also preserves more information about their multidimensional distribution than simple arithmetic summation. For instance, Patient 1 [Z = (3.9, 2.8, 2.7)]and Patient 2 [Z = (1.4, 1.73, 6.28)] had nearly identical summed differences (9.40 and 9.41, respectively), which would suggest a similar overall degree of asymmetry if direct addition were used. However, their OAD values were 5.51 and 6.66, indicating that these two patterns were not equivalent. Specifically, the asymmetry in Patient 2 was predominantly driven by extraocular muscle involvement, whereas the asymmetry in Patient1 was more evenly distributed across tissues. A similar contrast was observed between Patient 3 [Z = (1.7, 0.6, 8.5)] and Patient 4 [Z = (4.9, 6.18, 1.25)]. Although Patient 3 had a lower summed difference than Patient 4 (10.8 vs. 12.33), the corresponding OAD was higher in Patient 3 (8.69 vs. 7.99), reflecting the disproportionate contribution of extraocular muscle asymmetry. These examples illustrate that OAD is not simply a composite score of asymmetry, but a distance-based metric that captures both the magnitude and the multidimensional structure of inter-orbital differences. Such a property may enhance its value in identifying clinically meaningful orbital imbalance associated with diplopia in TAO.

In particular, ROC curve analysis comparing the discriminatory performance of the predictive models further supported the relatively stronger performance of OAD. The age-adjusted OAD model showed the highest discriminatory ability (AUC = 0.940), outperforming the dEMa+ age model (AUC = 0.890) and the TRAb + age model(AUC = 0.785). These findings extend prior work on orbital imaging in TAO by highlighting the potential clinical relevance of asymmetry-based quantitative imaging indices to functional involvement, particularly diplopia. When OAD, dEMa, and age were combined, the AUC increased slightly to 0.952, compared with 0.940 for the OAD + age model. This marginal gain suggests that dEMa contributes little additional discriminatory information beyond that already captured by OAD. Interestingly, when these variables were entered simultaneously into a multivariable conditional logistic regression model, none of the individual predictors remained statistically significant. One possible explanation is multicollinearity, as OAD incorporates SUVmax-derived asymmetry information from the extraocular muscles and is therefore likely to overlap substantially with dEMa, making it difficult to disentangle their independent effects. Another contributing factor may be the limited sample size, which may have reduced the statistical power of the multivariable model. Despite this, the consistently strong performance of OAD across age-adjusted models supports its potential utility as a robust imaging marker for diplopia risk assessment in TAO.

Because this study used a matched case–control design and conditional logistic regression, internal validation was additionally performed using leave-one-pair-out cross-validation to preserve the matched-pair structure. Under this validation framework, the OAD + age model maintained the best pair-level discriminatory performance, correctly ranking the diplopia case above the matched control in 81.8% of held-out pairs. By comparison, the dEMa + age and TRAb + age models correctly ranked 72.7% and 68.2% of held-out pairs, respectively, whereas the OAD + dEMa + age model did not improve upon the performance of OAD + age alone. These findings further support the relatively stronger discriminatory value of OAD compared with dEMa and TRAb. Beyond its discriminatory performance, OAD may provide complementary imaging information in the assessment of functional involvement in TAO, alongside established imaging approaches such as STIR MRI for orbital inflammatory evaluation (13).

This study has several limitations. First, the sample size was relatively small (22 matched pairs), which limited statistical power and the precision of the estimated associations; therefore, the observed associations of OAD and dEMa with diplopia should be interpreted with caution. Second, although internal validation was performed, external validation in an independent cohort was not available. Accordingly, the proposed cutoff values should be considered exploratory and require prospective validation in an independent cohort before OAD can be recommended for clinical risk stratification. Third, diplopia was identified from routine clinical assessment and medical records rather than standardized orthoptic testing, and subtle or posture-compensated diplopia may therefore have been underrecognized.

## 5 Conclusion

Taken together, our findings suggest that inter-orbital inflammatory asymmetry is associated with diplopia in TAO. OAD, a novel ^99m^Tc-DTPA SPECT/CT-based quantitative imaging marker, was significantly associated with diplopia and showed relatively strong discriminatory performance. Further studies in larger prospective cohorts with independent external validation are warranted to confirm these findings and to clarify the clinical utility of OAD in combination with other clinical and imaging markers before it can be considered for clinical risk stratification.

## Data Availability

The original contributions presented in the study are included in the article/**Supplementary Material**. Further inquiries can be directed to the corresponding author.
