# Supplementary material for: Orbital asymmetry degree: a potential 99mTc-DTPA SPECT/CT imaging marker for diplopia in thyroid-associated ophthalmopathy
Source: Front Endocrinol (Lausanne). 2026 Aug 13;17:1871573. doi: 10.3389/fendo.2026.1871573 (PMC13518127; doi:10.3389/fendo.2026.1871573)
Supplement: Supplementary file 1 [file DataSheet1.docx]

Supplementary Material

## Supplementary Figures and Tables(2 supplementary figures and 3 supplementary tables)

## 1. Supplementary Figures

**Supplementary Figure 1.** **Representative orbital ^99m^Tc-DTPA SPECT/CT findings in a bilateral TAO patient with diplopia** A 65-year-old woman with bilateral thyroid-associated ophthalmopathy presented with diplopia for 1 year. Orbital ^99m^Tc-DTPA SPECT/CT images are shown in axial (A), coronal (B), and sagittal (C) views. In the axial image (A), markedly increased tracer uptake is seen in the right medial rectus muscle compared with the left side (red arrows). The coronal image (B) demonstrates substantial interocular asymmetry in tracer uptake involving multiple extraocular muscles. The Clinical Activity Score (CAS) was 4 in both eyes. The corresponding Z(1.7, 0.6, 8.5), and the OAD value was 8.68.

**Supplementary Figure 2. Representative orbital ^99m^Tc-DTPA SPECT/CT findings in a bilateral TAO patient without diplopia.**

A 37-year-old woman with TAO presented without diplopia. Orbital ^99m^Tc-DTPA SPECT/CT images are shown in axial (A), coronal (B), and sagittal (C) views. Mildly increased tracer uptake was observed bilaterally in the extraocular muscles and lacrimal glands on the axial and coronal images (red and yellow arrows, respectively); however, the uptake pattern was symmetrical, without obvious interocular differences. No focal asymmetric increase in tracer uptake was detected in the orbital soft tissues. CAS was 1 in both eyes. Z=(0,2.1,0.5),and OAD=2.158，which were consistent with a non-diplopic presentation.

**2.Supplementary Tables**

**Supplementary Table 1. Supplementary comparison of extraocular muscle SUVmax and CT-based extraocular muscle thickness measurements between the case and control groups.**

| Variable | Case group(n=22) | Control group(n=22) | *P* value |
| --- | --- | --- | --- |
| $\text{SUVmax}\text{\_}\text{EM}\text{,}\text{left}$ | 1.053 ± 0.272* | 1.066 ± 0.357* | 0.894 |
| $\text{SUVmax}\text{\_}\text{EM}\text{,}\text{rig}\text{h}\text{t}$ | 1.030 (0.908, 1.185) | 1.035 (0.883, 1.118) | 0.987 |
| Mean Emt(L) | 3.318 (2.873, 3.833) | 3.054 (2.768, 3.420) | 0.048 |
| Mean Emt(R) | 3.461 (3.094, 3.668) | 3.068 (2.735, 3.527) | 0.079 |
| Absolute inter-orbital difference in mean extraocular muscle thickness (mm) | 0.344 ± 0.232* | 0.426 ± 0.303* | 0.243 |

^SUVmax_EM,left and SUVmax_EM,right denote the extraocular muscle SUVmax values for the left and right orbits, respectively.^

^Mean Emt (L) and mean Emt (R) represent the mean extraocular muscle thicknesses of the left and right orbit, respectively.^

^Absolute inter-orbital difference in mean extraocular muscle thickness was defined as the absolute value of the difference between left and right mean extraocular muscle thicknesses for each patient.^

^*Data are presented as mean ± standard deviation ,or else presented as median (interquartile range)^

**Supplementary Table 2. Age-adjusted conditional logistic regression analysis of CT-based mean extraocular muscle thickness of the left and right orbits in patients with TAO**

| Variable | Age-adjusted OR | 95%CI | *P* Value |
| --- | --- | --- | --- |
| Mean Emt(L) | 0.676 | 0.249~1.837 | 0.443 |
| Mean Emt(R) | 0.799 | 0.278~2.292 | 0.676 |

^Mean Emt (L) and mean Emt (R) represent the mean extraocular muscle thicknesses of the left and right orbit, respectively.^

**Supplementary Table 3. Age-adjusted conditional logistic regression results for the combined OAD + dEMa model in patients with TAO.**

| Model | Variable | OR | 95%CI | *P* value |
| --- | --- | --- | --- | --- |
| OAD+ dEMa+ Age | dEMa | 1.307 | 0.708~2.411 | 0.392 |
|  | OAD | 1.912 | 0.945~3.87 | 0.071 |
|  | Age | 0.296 | 0.084~1.045 | 0.059 |
